# Strategies for Promoting Healthy Nutrition and Physical Activity Among Young Children: Priorities of Two Indigenous Communities in Canada

**DOI:** 10.1093/cdn/nzz137

**Published:** 2019-11-28

**Authors:** Gita Wahi, Julie Wilson, Richard Oster, Patricia Rain, Susan M Jack, Joel Gittelsohn, Sujane Kandasamy, Russell J de Souza, Cindy L Martin, Ellen Toth, Sonia S Anand

**Affiliations:** 1 Department of Pediatrics, Faculty of Health Sciences, McMaster University, Hamilton, Ontario, Canada; 2 Department of Health Research Methods, Evidence and Impact, Faculty of Health Sciences, McMaster University, Hamilton, Ontario, Canada; 3 Six Nations Health Services, Six Nations of the Grand River, Ohsweken, Ontario, Canada; 4 Department of Medicine, University of Alberta, Edmonton, Alberta, Canada; 5 Maskwacis Health Services, Samson Cree Nation, Maskwacis, Alberta, Canada; 6 School of Nursing, Faculty of Health Sciences, McMaster University, Hamilton, Ontario, Canada; 7 Center for Human Nutrition, Department of International Health, Johns Hopkins Bloomberg School of Public Health, Baltimore, MD, USA; 8 Department of Medicine, Faculty of Health Sciences, McMaster University, Hamilton, Ontario, Canada

**Keywords:** Indigenous, child health, health promotion, nutrition, physical activity

## Abstract

**Background:**

Indigenous people in Canada carry a disproportionate burden of obesity and obesity-related diseases compared with non-Indigenous Canadians, which could be related to intergenerational trauma exposures. Implementing effective health promotion strategies to improve nutrition and physical activity behaviors during early childhood could be a strategy to mitigate the burden of intergenerational trauma exposures that have the potential to impact the trajectory to obesity and related complications throughout the lifecycle.

**Objectives:**

The aim of this study was to support 2 Indigenous communities in identifying priorities and strategies for promoting healthy nutrition and physical activity for young children.

**Methods:**

Using a formative approach, we conducted a 2-phase study that started with 2 community engagement workshops (*n* = 37 participants), followed by a qualitative descriptive study. In this latter study, in-depth interviews were conducted with a purposeful sample of 23 community parents, health care providers, and traditional knowledge holders. Data from both study phases were analyzed and synthesized using conventional content analysis.

**Results:**

To promote healthy nutrition and physical activity among young children living in Indigenous communities, it was identified that the primary pathway to health and well-being must prioritize the integration of knowledge about Indigenous ways of life including traditional Indigenous foods and physical activities. Participants also identified individual/family and community/contextual factors that ultimately influence the nutrition and physical activity of children in their communities.

**Conclusions:**

Informed by this formative study conducted to better understand community members’ strategies for healthy eating and physical activity for young children, we argue for the continued recognition of the unique Indigenous context, incorporating the history of inequity and injustice and looking toward Indigenous-led interventions that incorporate this history and ways of life as solutions in the future.

## Introduction

Since the late 1990s there has been an exponential rise in global rates of noncommunicable diseases (NCDs) (1). There is emerging evidence suggesting that the propensity to develop NCDs and poor health outcomes is strongly influenced by adverse childhood experiences (ACEs) (2–4). Common ACEs include exposure to family violence, child maltreatment, mental illness, and, in the context of Indigenous people of Canada, multiple historical traumas (5, 6).

Indigenous people in Canada include 3 distinct groups: First Nations, Métis, and Inuit. Among these communities, individuals have endured historical traumas perpetrated by social, political, and religious institutions, including colonization and being removed from their homes and isolated in residential schools. These traumas have resulted in the loss of traditional lifestyles, language, and significant challenges in adapting to the encroachment of the dominant society, which in turn has led to substantial health inequities (7). A large proportion of Indigenous people in Canada live with limited resources, and methods of coping with this adversity can include, among other pathways, adoption of risky lifestyle behaviors including consumption of poor diets and low physical activity (4). Indigenous people in Canada, including children and youth, carry a disproportionate burden of obesity and related diseases including type 2 diabetes and cardiovascular diseases compared with non-Indigenous Canadians (8–10). Evidence suggests that the development of obesity and related conditions is rooted in early life experiences (11). Therefore implementing effective health promotion strategies to improve nutrition and physical activity behaviors during early childhood could be a strategy to mitigate the burden of intergenerational trauma exposures and the health impact across generations, and impact the trajectory of obesity and related complications throughout the lifecycle.

Successful health promotion strategies must be pragmatic, culturally tailored, and consider contextual facilitators and barriers to implementation (12). In obesity prevention programs, healthy behaviors include limiting the consumption of energy-dense, nutrient-poor foods, minimizing sedentary behaviors, and staying physically active. Traditional Indigenous knowledge and beliefs have endured in Canada despite historical traumas, and this tremendous resiliency emphasizes the central place of traditional teachings and culture in the design of health interventions (13). There is limited evidence for obesity prevention interventions with young Indigenous children in Canada (14, 15). Our partnership in formative work provides opportunity for Indigenous communities to shape future interventions and tailor them for cultural and geographical context (16).

This study was built on a longstanding partnership between academic researchers from McMaster University and the University of Alberta and 2 Indigenous communities in Canada, Six Nations of the Grand River, Ontario, and Pigeon Lake, Alberta (8, 12, 17–19). The partnership between the Six Nations community and academic researchers includes ongoing work on a birth cohort study to understand the cardiometabolic risk factor profile of Indigenous women and infants, and ways to promote health outcomes (17). The overarching goal of this project, which included community engagement workshops as well as a descriptive qualitative study, was to identify and understand community-identified strategies for the primary prevention of childhood obesity among young Indigenous children (<5 y) in Canada that could be used to inform the development of future health promotion interventions. Using a previously established formative research approach to intervention development, we identified community priorities for nutrition and physical activity for young children and families, and community strategies to prevent obesity and promote health (16).

## Methods

### Setting

The Six Nations Reserve is situated in Brant County, Ontario, took its present form of 20,000 ha in 1847, and is home to >12,000 Six Nations people (8). Pigeon Lake Reserve covers 1921 ha and is situated in central Alberta, bordering Wetaskiwin County, with a population of 429 according to the Statistics Canada 2016 Census (20, 21). The Pigeon Lake Reserve is shared between the 4 communities that comprise the community of Maskwacis: Ermineskin Cree Nation, Louis Bull Cree Nation, Samson Cree Nation, and Montana Cree Nation. Despite differences in the size of the population of the communities, both are similar distances to large Canadian cities and each has a community health center on reserve.

### Study design

We used a formative research approach for the initial stages of intervention development (16). Benefits of formative research include its inclusivity in producing an intervention that is both culturally and geographically appropriate to a community (16). Formative research methods are typically conducted with both qualitative and quantitative methodologies and can use multiple approaches to data collection and community input (17, 22). We conducted a 2-phase study between September 2015 and June 2017. In phase 1, we held community engagement workshops in each community focused on nutrition and physical activity for children aged <5 y. The goals of these workshops were to: *1*) identify priority nutrition and physical activity issues for community members who live and work with young children and families; *2*) discuss key principles that would inform how to address the priorities; and *3*) identify key stakeholders for the next research phase. The purpose of phase 2, a qualitative descriptive study, was to deepen our understanding of the themes that arose during phase 1, and to continue to identify and document community priorities and strategies to address this important health issue (23). Findings from phase 1 that informed the protocol for phase 2 included strategies for purposeful sampling and key concepts to explore within the context of semistructured, in-depth interviews. An audit trail was kept detailing all decisions made throughout the study to ensure that the study's conclusions represent the data.

### Ethics

Approval was received from the Hamilton Integrated Research Ethics Board, University of Alberta Research Ethics Board, the Six Nations Council Research Ethics Committee, and partners within Pigeon Lake Reserve. The study followed guidelines to ensure informed consent, study governance, and publications in keeping with the Tri-Council Policy Statement 2, Chapter 9 on Research Involving the First Nations, Inuit, and Métis Peoples of Canada (24) and OCAP® (ownership, control, access, and possession) principles (25). Participants provided written informed consent prior to individual and group interviews, which included follow-up contact if needed.

### Phase 1: Community engagement workshops

Members of the research team collaborated with a Six Nations of the Grand River community leader who was a partner on the study to plan the community engagement workshops. The community leader identified 3 community stakeholders with extensive experience working with community families with young children, who guided the researchers on how to successfully organize and facilitate a community engagement workshop. We planned 2 workshops: 1 in Six Nations of the Grand River and another in Pigeon Lake. To participate in the workshops we invited community members who had experience in any of the following areas: child health, community programs, parenting, health promotion, Indigenous knowledge keepers, or elders.

In September 2015, we held the initial community workshop at Six Nations of the Grand River. In total, 21 people participated including representatives from Six Nations health services, local schools, and daycares as well as community members including elders and academic researchers. The initial workshop was cofacilitated by an academic researcher (JG) and a community health leader (JW). In January 2016, the second community workshop was held in Pigeon Lake following a similar protocol. A total of 16 participants, including parents, elders, academic researchers, and representatives from health services joined the meeting held at the Pigeon Lake Health Centre. An academic researcher present at the Six Nations workshop (GW) cofacilitated the workshop in Pigeon Lake with an academic researcher who works with the community (RO). For both workshops, after a brief introduction of the objectives for the day, participants introduced themselves and discussed their experiences with young children and health as well as their goals for the day. The agenda for the community workshop included: introduction of all participants; identification of nutrition and physical activity priorities for young children; identification of community resources for young children; facilitators and barriers to accessing resources; and identification of potential solutions. The prioritization activities with the initial topics chosen were based on previous community workshops (22), although the agenda was flexible and changed based on input from participants.

#### Analysis plan

For the workshop, facilitators addressed each agenda item, using techniques similar to those described by Gittelsohn et al. (22) in the development of chronic disease prevention programs with Inuit communities. After each topic was introduced, participants brainstormed and listed items specific to each topic. A member of the team wrote all the ideas on a flip chart in a list format. The results were posted on the walls of the workshop room. Participating community members then identified their priority issues within each category through a process of voting, by placing a sticker next to their preferred option. The items identified as high priority were those with the highest number of votes. The results of the lists with prioritization from participants as well as written notes summarizing the discussion during the workshops were returned to all participants within two weeks of the workshop.

### Phase 2: Qualitative descriptive study

Building on the findings from the phase 1 community engagement workshops, the principles of fundamental qualitative description informed all sampling, data collection, and analytic decisions in phase 2 (26). Qualitative description is used to describe a phenomenon with a low amount of inference by the researcher and staying close to the language and words of the participants (26). The academic researchers on the team were non-Indigenous, therefore this methodology allowed prioritization of the voice and ideas of the community members to be highlighted and to direct areas of priority and need with respect to the topic of research.

The purposeful sample we invited to participate in this phase of the study included: *1*) community members providing health care services and/or health promotion programming; *2*) current parents/caregivers of young children (aged <5 y); and *3*) community members with Indigenous knowledge, for example, elders. At the onset of this phase of the study, we invited key informants identified from the phase 1 workshop with a high degree of knowledge about the topic. Using snowball sampling, we identified community members from this initial sample who could speak knowledgeably about emerging themes (27). Sampling continued until saturation—the point when information from data sources does not contribute new information to the emerging themes—was reached (27). The research group decided when saturation had been reached through group discussion of emerging themes. To achieve credibility, member-checking interviews with 5 participants were completed and included review of data and emerging themes. A combination of individual and group interviews provided triangulation of data sources (28).

We interviewed 23 participants (Six Nations *n* = 13, Pigeon Lake *n* = 10). The participants included community members involved with health promotion programs (*n* = 14); mothers with young children who lived in the community at the time of recruitment (*n* = 5); and elders (*n*  = 4). Each interview was a single, face-to-face, one-to-one, in-depth, semistructured interview of ∼60 min. In Six Nations of the Grand River the interviews were facilitated by an academic researcher and pediatrician (GW) who has worked with the community since 2011. Interviews in Pigeon Lake were facilitated by either an academic researcher who has worked with the community since 2013 (RO) or a community research associate. The interview guide was directly informed by the findings summarized from the community workshops and revised after the first few interviews. It included questions on nutrition and physical activity priorities for families with young children similar to the workshops; it also included questions on culture and traditional knowledge in health promotion, as well as discussion of facilitators and barriers to health promotion in follow-up to discussion from the workshops. To maintain neutrality and limit bias, a reflexive journal was kept by the primary academic researcher (GW), with specific attention to her role as a non-Indigenous researcher. We audio-recorded and transcribed all interviews verbatim with personal identifying information omitted. Interview manuscripts were imported into NVivo-11 software (QSR International Pty Ltd).

#### Analysis

The principles of conventional content analysis guided the coding and synthesis of these data, and included processes of open, focused, and thematic coding for the purposes of creating linkages (29). Two team members (GW and SK) coded all the data separately and then met to discuss and resolve differences. A member of the community participated in and reviewed the development of the coding schema (JW), and in Pigeon Lake a team member (RO) reviewed the coding schema with a community member with knowledge of child health issues.

## Results

The overarching theme generated through both research phases, informed by the experiences of knowledge holders, was the following: *Traditional Indigenous ways of life are the primary pathway to health and well-being for young children and families*.

Participants described that traditional, Indigenous ways of life were promoted through traditional knowledge and teachings and that there was an important connection between traditional ways of life and health. This is how one community member described the intersection between tradition, culture, and health: *“Traditional health is based on our teachings and our customs, and knowledge that has been passed down. Everything that has been in the past, that has guided our people for generations, it's still valid today.” [P1]* This process of passing on traditional knowledge, practices, and worldviews from one generation to the next was described by participants as being important and the responsibility of all community members. Further, successful understanding and engagement was perceived to be achieved when this form of cultural continuity occurs in multiple settings including home, schools, and the community at large. Multigeneration engagement in traditional activities was seen as fundamentally instrumental to promoting the physical, mental, and spiritual well-being of young children. Having an appreciation of past practices, and then recognizing similar activities and strengths in a current generation of children by parents, was seen as a valuable process, as one parent shared:

*When you look back many, many years ago and you really try to visualize what life was like for people. Hunting, the little boys would be 5 or younger and they would go out with their dads roaming in the fields. And I find my little one has a lot of that inside of him, where he likes to explore, and he's got sticks. [P10]*

Participants identified strategies to increase knowledge and understanding of traditional practices including: *1*) learning through personal family teachings; *2*) integration of traditional knowledge within community programming; *3*) embedding traditional knowledge and skills into school curricula; and *4*) teachings from elders. Many participants described the high value families placed on traditional Indigenous teachings, which were also an important element for healthy child development; this is illustrated by one mother's description of her family experience of learning traditional activities: *“I think when the parents have that in their mind, body, and spirit, the First Nations teachings then that really gets passed on because then you're encouraging them.” [P10]* Along with the family unit, community-led programs were described as an area of strength and a source of traditional knowledge and teachings, and included community gardens, baby food–making classes, cooking classes, and other health promotion community initiatives. Further, schools and daycares were considered important venues for passing on traditional knowledge to children; participants in one community discussed an example of a local elementary school committed to teaching Indigenous language, serving traditional foods, and providing opportunities for culturally relevant physical activities. This example was a community-led model of culture integrated into daily school teachings, including experiences with traditional foods, language, hunting, and gathering for the next generation. Finally, all community members spoke in high regard of elders in the communities and the important role they have as holders of traditional knowledge, with their integral role in the physical and mental well-being of communities.

### Indigenous ways of life include traditional foods and physical activities

Across both phases of the project, multiple conversations were held about the types of food most commonly consumed and the physical activities encouraged for children in the 2 communities. Indigenous ways of life including traditional knowledge and teachings are considered synonymous with healthy food choices and participation in regular physical activity in both communities. This link was further exemplified in the workshop activities, where participants were asked to prioritize the foods consumed as well as the types of physical activities young children participated in. The following foods were identified by participants as “healthy” foods consumed by children in the community: “water, fish, white corn, wild meats, seeds, berries, homemade soups, and vegetables” (**Table 1**). The discussion that arose following this prioritization activity included observations that foods on this list were more often whole ingredients compared with ready-to-eat foods. Further, foods on the lists were identified as part of a traditional Indigenous diet for each community. In comparison, community members were acutely aware of the frequency and extent that many local children also regularly consumed processed, sugar-laden foods in their daily diets. The most common food sources that were labeled by participants as “unhealthy” included “pop, fast food, pasta, sugary foods, grains (e.g., cereal, pastries, and bread), and other sweet drinks” (**Table 2**). Physical activities considered important and desired included traditional activities such as hunting or traditional games that were connected to the land and the outdoors. During the workshop, participants brainstormed different types of activities that should be promoted to young children. The most common types of physical activities that were prioritized by participants included: “walking, running, school sports, winter play/sports, gardening, swimming, traditional dancing, hunting, and picking food” (see **Table 3** for priority physical activities for each community). In discussion at the workshop many of the participants described the characteristics of ideal opportunities for physical activity for young children: such activities would have minimal to no cost and be accessible in the community setting.

**TABLE 1:** List of 10 most common foods that each community prioritized as healthy for young children (aged <5 y): phase 1

	Community A		Community B	
Rank	Food item	No. votes	Food item	No. votes
1	Water	20	Neckbones	9
2	Fish	13	Berries	8
3	Whole grains	12	Moose	8
4	White corn	11	Subway	7
5	Wild meat	11	Blender foods	5
6	Wild rice	11	Tea	5
7	Seeds	10	Vegetables	4
8	Strawberries	9	Roasts	4
9	Deer meat	8	Steak	4
10	Homemade soups	8	Potatoes	4

**TABLE 2:** List of 10 most common foods that each community prioritized as unhealthy for young children (aged <5 y): phase 1^1^

	Community A		Community B	
Rank	Food item	No. votes	Food item	No. votes
1	Pop (soft drinks)	17	Take-out	13
2	Fast food	17	Pop	12
3	Pasta	17	Chips	12
4	Sugar cereal	12	Flour	9
5	French fries	11	Alcohol	8
6	Pizza	11	Raw noodles	6
7	GMO foods	9	Drugs	6
8	Poutine	7	Salt	6
9	Pastries	6	Candies	5
10	Bread	6	Chicken burgers	5

1 GMO, genetically modified organism.

**TABLE 3:** List of 10 most common physical activities that each community prioritized as important for young children (aged <5 y): phase 1

	Community A		Community B	
Rank	Activity	No. votes	Activity	No. votes
1	Walking	19	Powwow dancing	16
2	Running	16	Going for walks	11
3	School sports	14	Playing outside	9
4	Winter play/sports	11	Trampoline	7
5	Gardening	10	Hunting	7
6	Interactive/imaginary play	10	Swimming	6
7	Swimming	9	Dancing	5
8	Playing	9	Games	5
9	Circle games	9	Skating	5
10	Picking food	8	Beading	5

### Factors influencing young children's dietary and physical activity patterns of behavior

Through data triangulation and synthesis of emergent categories, we identified individual/family and community/contextual factors that influence the uptake of healthy nutrition and physical activity for young children living in these communities.

#### Individual and family factors

The individual and family factors contributing to dietary and physical activity patterns of behaviors were significant, and included: economic factors, physical factors, and individuals’ knowledge and skills. Participants described that unless these factors were addressed, families would have difficulty in making healthy food and regular physical activity a priority. Economic factors such as income were particularly a concern for participants, and low income and its connection with food insecurity was described by participants. A community health care provider described her experience with clients and food insecurity, citing a lack of reliable access to a sufficient quantity of food. Specifically, food insecurity would come up in indirect ways such as not having other basic items for young children or through how food provisions, such as food baskets, were received:

*If there is food insecurity it can be missed, especially if people don't say anything. I have had some recently, that don't have money for a car seat when baby is born. So, helping them through that, but there is a lot that don't say it. But sometimes we do home visits, or through talking to them, and how they receive the food basket is a really good indicator. [P6]*

A family's level of income was also perceived to limit traditional food consumption because these sources of nutrition were perceived to be more expensive compared with processed, energy-dense convenient foods that were more conveniently located in their neighborhoods. Further, in one community, participants described that local food banks commonly stocked foods that were energy-dense and of poorer quality, which made it difficult to avoid these nutrition sources for families in need. One health care professional further illustrated the additive impact of these barriers and shared that, “[community programming] *to think of things for a family that might say, ‘I don't have the transportation, I don't have the money, I don't have the resources.’” [P3]* For the impact of finances on physical activity many participants described a minimal cost associated with many activities in their communities such as walking, running, and school sports (Table 3).

The physical factors that were identified and perceived to limit healthy diets and regular physical activity for children and family were time and transportation. Participants described that families with young children have busy schedules that prevented home preparation of meals and meal planning. Physical activity was also limited by time, and one community member described her approach to physical activity community programming that considers the factors that influence active play:

*A lot of the families that I work with the moms has two jobs or it's just really hard to find that time when the child is available and mom is not making dinner or bathing another child, or anything of that sort, and so that is why we thought unstructured was the best way, given those five minutes in the middle that you have that you can play with the child. Because taking them for programming again, is expensive and you need transportation. So just low tech, anything at home that you can play with, we try to encourage. [P4]*

Transportation was described as impacting families in the communities and their participation in health promotion programing; one community member described transportation as a barrier to access and how they try to address it: *“Always transportation. Our program is getting a van, which will help a lot because transportation is a big thing, it's not a very walkable community, other than this area.” [P7]* In the discussion of the barriers of time and transportation participants described community-led solutions to support families.

Participants also described individual and family strategies for engaging in healthy food consumption and encouraging physical activity. One solution described by participants was community programs aimed at increasing individuals’ nutrition knowledge and food skills. Participants noted that there were many community programs run with the intention of addressing gaps in individuals’ or families’ knowledge and skills pertaining to food preparation: for example, events in community kitchens were noted to be useful in one community. Further, it was also described that community gardens were a source for alternative foods once the perceived barriers of time and lack of knowledge were addressed. One community member described current successful community programs that promoted healthy food consumption:

*We have done some successful cooking classes, several a year in fact, with great attendance. There are initiatives within the community and the health centers to teach people how to eat more healthy meals on the go. So, there's, and that's been a huge success. I am not 100% aware of what all the communities do, but I know that there's initiatives that are going on as we speak for community gardens. [P13]*

Another positive strategy that engaged families was community-run health promotion programs. Many participants described the important contribution of a strong family unit to a healthy community, and prioritizing families was considered an essential pathway to promoting health for the community as a whole. In planning community events and initiatives many participants discussed attending to the needs of the family unit, such as child care, meals, and activities for all members. Participants described the variety of community-led programs that were family focused in promoting healthy lifestyles. They highlighted the key elements of successful programs, including: *1*) promoting a family-friendly atmosphere; *2*) providing child care or age-appropriate activities for all participants; *3*) providing healthy food for participants; *4*) respecting that families are busy and developing programs with this in mind; *5*) incorporating community initiatives such as community gardens; and *6*) addressing the unique needs of families that can prevent them from attending community programs, such as family meals, transportation, and admission costs.

#### Community and contextual factors

Woven through all participants’ narratives, there was an awareness and deep consideration of how community and contextual factors, specifically geographic, historical, and cultural factors, influence nutrition and physical activity within the community. Geographic factors included commonly identified barriers such as community safety and environmental contamination. Participants described personal safety as barriers to participation in physical activity programs; for example, a participant described how the physical environment can deter a family from participating in physical activity: “*We are hoping for a safer community as a whole, so that a lot of the moms that do have these fears are able to go out and do things with their kids. You don't see a lot of people walking on the road. It's not safe.” [P3]* The contamination of the physical environment was a barrier to traditional food consumption described by participants. Community members described high levels of environmental contamination and the negative impact on food sources, especially fishing in local lakes. One community member described how they perceive this environmental contamination and its effect on food sources: “*When I was little, we lived on fish and wild meat. And vegetables, wild berries. But when they came here, it's different. Some still do all those things, yeah. But we can't eat the fish from here.” [P22]* The land and its attributes were considered very important and their roles must be recognized and acknowledged.

A clear and overarching theme throughout the study was the importance of understanding context, specifically the impact of colonization in shaping the life course of community members. One community health care provider described how historical contextual factors can impact connection to culture.

*“It's the shaming around the identity of the culture. Like people didn't want to be associated as being Indigenous or First Nations, they didn't want anyone to know because of the influence of colonization.” [P2]*

Connection to culture, family, and socioeconomic barriers are all impacted by historical traumas, and this context is essential for understanding the data. One community member described how loss of connection to Indigenous traditions and teachings has impacted traditional activities: *“The knowledge to go hunting, fishing, we need more of that, ‘cause these life skills are part of our culture and we weren't allowed to do that, so it's not being passed down.” [P1]* Further, in striving to achieve health, community members discussed the need to understand the impact of colonization and its role with their present-day lives. Participants described eating traditional foods and participating in traditional physical activities as strategies to promote health. One community member described the importance of bringing traditional knowledge into current-day practice:

*A lot of our spiritual practices and a lot of our traditional knowledge of medicine, a lot of those things is what fell through the cracks when we came here and, so I think that's what's really impacting our community now and where children are feeling disconnected. People are making a move back to growing their own food, but we've come so far from there. [P5]*

Consuming foods based on a traditional diet from community-led health initiatives was considered a model for a healthy diet. This is demonstrated by a community health care provider's description of the importance of traditional foods to health: *“So looking at food, I think how food connects to our spirit. And a lot of us have disconnected that food to our spirit and that linkage.” [P2]* The important context of tradition and culture is a vital factor that must be considered in any programs and interventions for healthy nutrition and physical activity promotion.

## Discussion

This is the first study to describe the priorities and strategies of community members for promoting both healthy nutrition and physical activity among young Indigenous children in Canada. Our findings highlight that community members prioritize traditional Indigenous ways of life including traditional foods and physical activities, considering these the most important factors for promoting health for young children and families. Our study furthers emphasizes critical pieces for understanding and context, specifically the importance of cultural continuity and an overarching theme woven through the study—the importance of the distal factors that pervade health inequities, specifically the social determinants of health and historical traumas including colonization.

The intent of the workshops was to generate concrete examples and consensus ideas generated by community members in the areas of healthy dietary intake and physical activity to provide strategies for young children and families in the community to optimize these aspects of their lives. The discussions of the workshop attendees were consistent with current global recommendations (30). In a socioecological model of health behaviors, these would represent proximal factors, which often reside with the individual and/or family. Interventions that only target individual-level factors and do not address the unique Indigenous context in Canada, including intergenerational traumas, are likely to fail (31). In comparison, interventions led and developed by Indigenous communities would, by definition, include an understanding of the Indigenous context and traditions and therefore have a greater likelihood of succeeding.

By addressing factors that promote traditional ways of life we were able to identify areas of strength within the community that facilitate healthy nutrition and physical activity, specifically various community programs and community-led initiatives that focus on culture, family, and the passion for educating children in the community. Participants prioritizing and describing the connection between physical activity for children to traditional, culturally based activities is similar to what Tang and Jardine (32) described with the Yellowknives Dene First Nation community. Led by the participant discussions, the workshops also addressed barriers to healthy lifestyles, which were also addressed in the qualitative study, specifically, economic, physical, and geographic factors. Similar barriers to traditional food systems were described by participants in 6 First Nations communities as described by McGregor et al. (33). Our findings are also in line with results of a previous study from one of the communities examining the contextual determinants of health behaviors in adults, which found an unfavorable built environment, specifically poor accessibility to active transportation, limited availability of healthy food, and poor safety (12). These barriers highlight the ongoing need to address community-level factors to promote healthy lifestyles in many Indigenous communities (31).

Cultural continuity is described as “the integration of people within their culture and the methods through which traditional knowledge is maintained and transmitted” (34). For this study the concept of cultural continuity facilitates the description of cultural connection as the pathway to health described by participants. Cultural continuity is protective and confers health benefits to Indigenous peoples in Canada (35, 36). In 1998 Chandler and Lalonde (36) examined cultural continuity as it related to youth suicide among First Nations communities in British Columbia, Canada. The authors identified 6 measures of cultural continuity (land titles, self-government, educational services, police and fire services, health delivery, cultural facilities) at the community level and described how each was independently related to and protective against youth suicide (36). A mixed methods study by Oster et al. (35) explored the concept of cultural continuity and its relation to rates of diabetes among First Nations Bands in Alberta, Canada. Cultural continuity was conceptualized as “being who we are” and very much connected to traditional Indigenous language. Oster et al. (35) went on to show that communities with more members speaking their traditional languages had significantly lower rates of diabetes. Our study adds to the literature because it shows another example of community-driven data that furthers the argument for culture as the central component of health and wellness as described by Indigenous community members including caregivers of young children. This is similar to work done by other Indigenous communities in Canada that highlights the importance of culture and its relation to nutrition and physical activity (32, 33). A logical extension of our work is a quantitative analysis to better understand the impact of culture continuity on healthy lifestyle factors, for example, nutrition and physical activity among families with young children in the community.

The strengths of this study included a rigorous methodological approach to sampling, data collection, and analysis. The triangulation of both data types and sources contributed to the overall credibility, or truth value, of the findings. Credibility of the findings was further enhanced by the participatory activities embedded in the data collection process, the partnerships between Indigenous and non-Indigenous researchers, each bringing unique worldviews and understanding of the issues under study to the analysis. The dependability of the findings was enhanced through a rigorous process of analysis, that again included a team approach to coding and interpretation. Limitations of the study included the small number of participants who were parents or caregivers and the paucity of fathers/male participants, who could have enriched the data. Further, although there was remarkable concordance in themes between the 2 communities, this study might not be applicable to all Indigenous communities in Canada, although similar themes have been established in other health areas and communities in Canada (32, 33, 35, 36).

Our study contributes to the mounting voices that call for cultural connection and continuity as a pathway to health and well-being for Indigenous peoples in Canada. What started as a formative study to better understand community members’ strategies for healthy eating and physical activity for young children transcended the individual-level factors and argues for the continued recognition of the unique Indigenous context incorporating the history of inequity and injustice, and looks toward Indigenous-led intervention that incorporates this history and traditions as possible solutions in the future.

## Notes

Supported by the Canadian Institutes of Health Research, funding reference number 137493.

Author disclosures: The authors report no conflicts of interest.
